# Lobular neoplasia: frequency and association with other breast lesions

**DOI:** 10.1186/1746-1596-6-74

**Published:** 2011-08-09

**Authors:** Douglas S Gomes, Débora Balabram, Simone S Porto, Helenice Gobbi

**Affiliations:** 1 Breast Pathology Laboratory, School of Medicine, Federal University of Minas Gerais (UFMG), Av. Professor Alfredo Balena, 190, Belo Horizonte, Minas Gerais; 30130-100, Brazil

**Keywords:** breast cancer, lobular neoplasia, ductal carcinoma *in situ*, columnar cell lesions

## Abstract

**Background:**

Using new molecular biology techniques, recent studies have implicated a common evolutionary pathway between lobular neoplasia, lobular carcinomas, and columnar cell lesions. Our aims were to assess the frequency of lobular neoplasia in a series of breast biopsies that were performed and examined in the same institution and to analyze the association between subtypes of lobular neoplasia and benign and malignant breast lesions.

**Methods:**

Cases were selected after reviewing archived pathological reports in the Breast Pathology Laboratory, School of Medicine of Federal University of Minas Gerais (1999-2008). Cases of lobular neoplasia were reviewed and classified as atypical lobular hyperplasia, ductal involvement by cells of atypical lobular hyperplasia, lobular carcinoma *in situ*, and pleomorphic lobular carcinoma *in situ*. Coexistence of lobular neoplasia with other breast lesions, including columnar cell lesions, invasive ductal carcinoma and invasive lobular carcinoma, was evaluated. The association between lobular neoplasia and breast lesions was analyzed by Fisher's exact test and chi-square test for linear trend.

**Results:**

We analyzed 5650 breast specimens, selecting 135 breast specimens (2.4%) that had a diagnosis of lobular neoplasia, corresponding to 106 patients. Hematoxylin and eosin-stained slides were available for 84 cases, 5 of which were excluded because they contained only "indeterminate" *in situ *lesions. Of the 79 remaining cases, columnar cell lesions were present in 78.5%, primarily with columnar cell changes without atypia (67.7%). Invasive carcinoma was present in 45.6% of cases of lobular neoplasia--a similar frequency (47.2%) as invasive ductal carcinoma and invasive lobular carcinoma. We noted a significant linear trend (p < 0.03) of a higher frequency of invasive carcinomas that were concomitant with lobular carcinoma *in situ *compared with atypical lobular hyperplasia. Invasive lobular carcinomas were associated with lobular carcinoma *in situ *in 33% of cases, compared with 2.8% of atypical lobular hyperplasia cases.

**Conclusions:**

Our findings confirm a frequent association between lobular neoplasia and columnar cell lesions, the majority of which lacked atypia. We also observed a greater frequency of invasive carcinoma, more commonly invasive lobular carcinoma, associated with more developed forms of lobular neoplasia (lobular carcinoma *in situ*).

## Background

Lobular carcinoma *in situ *(LCIS) was first described by Foote and Stewart in 1941, designated as such due to its cytological similarities with invasive lobular carcinoma (ILC): cuboidal and regular and harboring discohesive cells, often containing cytoplasmic vacuoles. LCIS was originally considered a precursor of invasive carcinoma due to its frequent association with invasive lobular carcinoma [1]. Subsequent epidemiological studies demonstrated that the risk of developing invasive lesions was not as high as expected, progressing slowly and forming in the ipsilateral and contralateral breast [2].

Other studies confirmed the indolent nature of LCIS; clinically, LCIS was considered a risk marker for invasive breast cancer. The consequent risk was proportional to the extent of disease and was evaluated, based on distention of the lobular units in the ducts that were affected by neoplastic cells [3,4]. Due to its indolent behavior, Haagensen *et al. *proposed replacing the term "lobular carcinoma" with "lobular neoplasia" to decrease the impact of the malignancy and the link to mortality that is associated with the term "carcinoma" [2].

Page *et al. *correlated the extension of lobular involvement and the risk of breast cancer, proposing a semiquantitative stratification method--designating lobular lesions in atypical lobular hyperplasia (ALH) for less extensive lesions and LCIS for more extensive lesions. A 4- to 5-fold relative risk of developing invasive carcinoma was observed for ALH lesions, whereas for CLIS, the relative risk was 8 to 11 times greater than the general population [4]. The ductal involvement by cells of atypical lobular hyperplasia (DIALH), also called pagetoid spread, carried an intermediate risk of developing carcinoma of 6.8-fold [5].

Although Page's classification has been used widely over the past 20 years, the latest World Health Organization (WHO) classification of tumors groups these lesions under lobular neoplasia (LN), without considering their development [6].

Recent molecular biology studies have revealed more about lobular neoplasia. Genetic similarities, such as the loss of chromosomal material on 16q and gains on 1q, have been observed in LN and other low-nuclear grade breast lesions. Similar genetic alterations were detected in columnar cell lesions (CCLs), low-grade ductal carcinoma *in situ *(DCIS), tubular carcinoma (TC), and ILC. These similarities suggest a common evolutionary pathway, in which low-grade precursor lesions progress to low-grade invasive and *in situ *carcinomas [7-9].

Columnar cell lesions coexist frequently with DCIS, and low-grade invasive carcinomas, particularly TC and ILC [10-13]. However, few studies have evaluated this association, based on the diagnosis of LN, in routinely removed breast specimens [14,15].

The aims of this study were to assess the frequency of LN in a series of breast biopsies that were performed and examined in the same institution and to analyze the association between subtypes of LN with benign and malignant breast lesions.

## Methods

We accessed the archives of the Breast Pathology Laboratory (BPL) of the School of Medicine of Federal University of Minas Gerais from August 1999 to December 2008, selecting all breast specimens with diagnoses of ALH, DIAL, LCIS, and pleomorphic LCIS. Cases of LN with original hematoxylin and eosin (H&E)-stained slides were reviewed by DSG and HG using a double-headed optical light microscope and included in the study. Breast biopsy specimens from the same patient were considered one case. Cases with only core needle biopsy specimens were excluded.

We used the histological criteria per Page *et al. *to classify ALH, DIALH, and LCIS [3-5]. LCIS was defined as complete involvement of the lobules by neoplastic cells, with greater than 50% of a lobule completely replaced and distended by neoplastic and monomorphic cells. ALH was defined as lobules that were partially distended by neoplastic cells, failing to meet the criteria for LCIS. DIALH was diagnosed when the ALH cells extended between the epithelial layer and the basement membrane of the terminal duct.

The criteria that we used to diagnose pleomorphic LCIS was described by Eusebi *et al.*--the same architectural pattern as LCIS but with larger nucleoli and nuclear pleomorphism [16]. Cases that harbored more than one subtype of LN were classified by the lesion with the greatest risk of developing carcinoma: pleomorphic LCIS > Classic LCIS > DIALH > ALH. The term "indeterminate *in situ *lesions" (IILs) or "mixed type lesions" was used to describe certain breast carcinomas *in situ*, in which the cytological or architectural properties and distribution deviated from the typical patterns, rendering it difficult, if not impossible, to determine whether the proliferation was lobular or ductal, based only on morphological criteria [17]. These cases were not included in our analysis.

The frequency of the association of LN was analyzed for the following diagnoses: CCL, per Schnitt and Vincent-Salomon [18], who divided the lesions into columnar cell change without atypia (CCC); columnar cell change with atypia (CCC with atypia); columnar cell hyperplasia without atypia (CCH); and columnar cell hyperplasia with atypia (CCH with atypia). The presence of *in situ *and invasive carcinoma was noted, as were their type and histological tumor grade. The tumors were classified per Page *et al. *and the American College of Pathology [19,20]. The Nottingham grading system was used for histological grading [21].

Statistical analysis was performed using SPSS (version 17.0, SPSS Inc, Chicago, IL, USA). Differences in mean age between LN groups was calculated by ANOVA, and the association between LN and breast lesions was analyzed using Fisher's exact test, χ^2 ^test, and χ^2 ^test for trend. The study was approved by the ethical committee of the UFMG.

## Results

During the study period, 5650 breast specimens from the same institution were analyzed. From the original reports, 135 breast specimens (2.4%) were diagnosed with a subtype of lobular neoplasia, corresponding to 106 patients, 21 of whom had 2 or more consecutive biopsies. H&E-stained slides were available for 84 patients, slides for 5 of whom were excluded because they contained only indeterminate *in situ *lesions. The frequencies of LN subtypes and the average patient ages are shown in Table 1. There was no significant difference in patient age between subgroups of patients with LN (*p *= 0.425).

**Table 1:** Frequency of subtypes of lobular neoplasia (LN) and mean age of patients

LN	n	%	Mean age (years) ± SD	
ALH	22	26.2	50.2	± 9.0
DIALH	25	29.8	50.2	± 9.7
LCIS	29	34.5	51.3	± 10.6
LCIS pleo	3	3.6	49.3	± 8.1
IIL	5	6.0	58.2	± 8.1
Total	84	100.0	52.0	± 9.7

ALH = atypical lobular hyperplasia; DIALH = ductal involvement by cells of atypical lobular hyperplasia; LCIS = lobular carcinoma *in situ*; LCIS pleo = pleomorphic LCIS; IIL = indeterminate *in situ *lesions. There was no difference in mean age among patients with different lesions (*p *= 0.425); n = number of cases; SD = standard deviation.

We observed a frequent association of LN with CCL (62/79 cases, 78.5%) and with most cases of CCC without atypia (42/62 cases, 67.7%). We observed a significant linear association (*p *= 0.03), wherein the frequency of LN tended to correlate negatively with the degree of atypical columnar lesions (Table 2). Twenty-three cases (29.1%) presented with coexisting LN, CCL, and invasive carcinoma (Table 2). Twenty cases (87%) comprised CCC or CCH without atypia, and 3 cases (13%) had CCC or CCH with atypia. There were no significant differences in the association of columnar lesions with or without atypia with regard to histological type and tumor grade of the invasive carcinomas. The coexistence of TC, LN, and CCL, reported by some groups as "Rosen's triad" [11], was observed in 1 case.

**Table 2:** Frequency of association between subtypes of lobular neoplasia and columnar cell lesions (CCL)

CCL	ALH		DIALH		LCIS		LCIS pleo		Total	
	n	%	n	%	n	%	n	%	n	%
CCC	14	22.6	13	21.0	15	24.2	0	0	42	67.7
CCH	4	6.5	3	4.8	2	3.2	0	0	9	14.5
CCC with atypia	1	1.6	3	4.8	4	6.5	1	1.6	9	14.5
CCH with atypia	0	0	0	0.0	1	1.6	1	1.6	2	3.2
Total	19	30.6	19	30.6	22	35.5	2	3.2	62	100.0

ALH = atypical lobular hyperplasia; DIALH = ductal involvement by cells of atypical lobular hyperplasia; LCIS = lobular carcinoma *in situ*; LCIS pleo = LCIS pleomorphic; CCC = columnar cell change; CCH = columnar cell hyperplasia; CCC with atypia = columnar cell change with atypia; CCH with atypia = columnar cell hyperplasia with atypia; n = number of cases. χ^2 ^test for trend: *p *= 0.03.

Moderate or usual ductal hyperplasia without atypia and atypical hyperplasia were present in 40% and 10.1% of 79 LN cases, respectively, but no significant difference in the association with LN subtypes was observed.

LN was associated with DCIS in 21.5% of cases, and high-grade DCIS correlated more often with LN (64.7% of cases). There were no cases that of concomitant DCIS and pleomorphic LCIS. Although there was no significant difference between LN subtypes, LCIS was most often associated with DCIS (47.1%) (Table 3). We noted 7 cases (8.9%) of LN and DCIS without concurrent invasive carcinoma--5 high-grade, 1 moderate, and 1 low-grade. Invasive carcinomas were present with LN in 45.6% of cases, with similar rates of association with invasive ductal carcinomas (IDCs) and ILC (47.2%).

**Table 3:** Association between subtypes of lobular neoplasia and histological grade of ductal carcinoma *in situ*

Histological grades of DCIS	ALH		DIALH		LCIS		Total	
	n	%	n	%	N	%	n	%
Low	0	0.0	2	11.8	2	11.8	4	23.5
Intermediate	1	5.9	0	0.0	1	5.9	2	11.8
High	2	11.8	4	23.5	5	29.4	11	64.7
Total	3	17.6	6	35.3	8	47.1	17	100.0

ALH = atypical lobular hyperplasia; DIALH = ductal involvement by cells of atypical lobular hyperplasia; LCIS = lobular carcinoma *in situ*; DCIS = ductal carcinoma *in situ*. There was no difference between groups.

With regard to cases of ILC, however, we observed a higher frequency of ILC that was associated with LCIS (33.3%) compared with DIALH (11.1%) and ALH (2.8%) (Table 4). No significant difference was noted in the link between histological grade of the invasive carcinoma and LN subtype in any group (Table 5).

**Table 4:** Association subtypes of lobular neoplasia and histological type of invasive carcinomas

Histological types	ALH		DIALH		LCIS		Total	
	n	%	n	%	N	%	n	%
IDC	4	11.1	4	11.1	9	25.0	17	47.2
ILC	1	2.8	4	11.1	12	33.3	17	47.2
Tubular carcinoma	0	0.0	1	2.8	0	0.0	1	2.8
Micropapillary	0	0.0	0	0.0	1	2.8	1	2.8
Total	5	13.9	9	25.0	22	61.1	36	100.0

ALH = atypical lobular hyperplasia; DIALH = ductal involvement by cells of atypical lobular hyperplasia; LCIS = lobular carcinoma *in situ*; IDC = invasive ductal carcinoma; ILC = invasive lobular carcinoma;. χ^2 ^test for trend: *p *= 0.03. There was no difference between groups.

**Table 5:** Association between subtypes of lobular neoplasia and histological tumor grade of invasive carcinomas

Tumor grade	ALH		DIALH		LCIS		Total	
	n	%	n	%	n	%	n	%
Low	1	2.8	3	8.3	12	33.3	16	44.4
Intermediate	2	56	4	11.1	9	25.0	15	41.7
High	2	5.6	2	5.6	1	2.8	5	13.9
Total	5	13.9	9	25.0	22	61.1	36	100.0

ALH = atypical lobular hyperplasia; DIALH = ductal involvement by cells of atypical lobular hyperplasia; LCIS = lobular carcinoma *in situ*; IDC = invasive ductal carcinoma; ILC = invasive lobular carcinoma. There was no difference between groups.

## Discussion

The frequency of diagnosis of lobular neoplasia in our study was 2.4% in a consecutive series of routinely removed breast specimens in a general hospital. The rate of LCIS ranges from 0.5% to 3.6% of breast specimens [2,3]. Because there are no obvious clinical or radiological features, the true incidence of LN in the general population is unknown [22,23].

The diagnosis of LN is typically related to an incidental finding on breast biopsies that are performed for other indications. With the increasing use of mammography, lobular neoplasia has been observed in association with microcalcifications in up to 40% of cases that are diagnosed by core needle biopsy [24]. Microcalcifications rarely form within LNs and they usually correlate with other benign or malignant breast lesions--the diagnosis of LN is most often incidental [23].

Columnar cell lesions (CCLs) comprise a spectrum of morphological alterations of the duct epithelial lining, acquiring a columnar cell appearance and involving variably dilated acini of the terminal duct lobular unit (TDLU) [25]. There has been recent, increasing interest in these lesions, because they are detected in up to 42% of the breast biopsies that are performed due to the presence of microcalcifications by mammography [26].

For instance, many terms have been used to describe CCLs, from "blunt duct adenosis" to "clinging carcinoma" [25,27]. Nevertheless, Schnitt and Vincent-Salomon's nomenclature and diagnostic criteria of CCL have been the most widely used [18], whereas in the most recent WHO guidelines, CCL was included under the term "flat epithelial atypia" (FEA) [6]. After the release of the WHO classification, Schnitt began referring to CCC and CCH with atypia as "flat atypia" [28].

CCLs have been linked to lobular neoplasia, low-grade DCIS, and invasive carcinoma. Further, similar genetic abnormalities have been found in CCC and CCH with atypia or FEA and the associated low-grade DCIS and invasive carcinoma. These findings have led to the reasonable conclusion that CCC and CCH with atypia are the earliest morphologically identifiable precursor lesions of low-grade DCIS and invasive carcinoma [8,15,25].

Yet, there are no prospective randomized trials, and few epidemiological studies with patients with only CCC and CCH with atypia have evaluated the prognosis of these lesions. Several studies, comprising a limited number of cases, demonstrated little or no risk for progression to invasive carcinoma [29-32]. Thus, there remains no consensus on the ideal treatment for these atypical lesions.

In our series, LN and CCL coexisted in 78.5% of cases, most often as mild forms of the spectrum of CCL--eg, CCC without atypia (67.7%). Our data are consistent with a recent study that examined 68 core needle biopsy specimens with a diagnosis of LN due to the excision of microcalcifications. The authors demonstrated an association between LN and CCL in 54% of cases, none of which presented with CCC or CCH with atypia after wide excision biopsy [15]. However, after analyzing 111 breast biopsies with LN but no other *in situ *or invasive carcinomas, Leibl *et al. *noted that LN was associated with FEA--ie, CCC and CCH with atypia in 86.5% of cases [33]. Our studies and other reports have observed a frequent association of CCL with LN, but they differ regarding the presence or absence of atypia.

There are many terms for LCC. Moreover, the WHO morphological definition of FEA is imprecise and does not describe the cytological and architectural features that are necessary for its diagnosis. In our study, all cases were reviewed by 2 observers, including a well-trained breast pathologist (HG). We used well-defined diagnostic criteria per Schnitt and Vincent-Salomon and noted fewer cases of CCC and CCH with atypia than what has been reported [10,11,33]. We believe that in many series and cases in our Breast Consulting Laboratory, FEA is being overdiagnosed, which could lead to the implementation of more aggressive treatments [34].

The frequency of invasive carcinomas that were associated with LN in our series was 45.6%, and we observed a similar frequency of ILC (47.2%) and invasive ductal carcinoma (IDC). However, when LN subtypes were analyzed separately, we observed a 4-fold higher frequency of IDC that was associated with ALH versus ILC and a greater link between ILC (33.3%) and LCIS compared with IDC. We also noted a 12-fold increase in the correlation between ILC and LCIS (33.3%) compared with ALH (2.8%).

Our data are consistent with a series of 775 cases of LN [14]. Bratthauer and Tavassoli stratified the LNs as "lobular intraepithelial neoplasias" (LINs) and evaluated the frequency of association between LIN subtypes (1, 2, and 3) and invasive carcinoma. The percentage of LIN 1 (equivalent to ALH) that was associated with invasive carcinoma was 14%, and 89% of these tumors were IDCs. In the patients with LIN 3 (equivalent to LCIS), the frequency of association with IDC and ILC was 23% and 86%, respectively. The authors concluded that the advance from LIN 1 to LIN 3 was linked to a 64% increase in the frequency of invasive carcinoma and a greater than 700% rise in the likelihood of ILC [14].

Our results corroborate other studies and suggest that lobular neoplasia is not only a risk indicator but also a nonobligate precursor of invasive breast carcinoma [23]. Invasive carcinomas that develop after a diagnosis of ALH are 3 times more likely to arise in the ipsilateral rather than contralateral breast [35].

Lobular neoplasia and ILC are detected together frequently in the same specimen and location of the tumor--in up to 90% of cases of ILC [10]. These lesions have similar immunohistochemical profiles, including the loss of expression of E-cadherin and β-catenin and the cytoplasmic localization of p120-catenin [36]. Invasive and *in situ *lobular carcinomas confer similar genetic gains and losses, often bearing the same mutations in the gene that encodes E-cadherin (*CDH1*) [7,37,38].

However, it is unknown why LCIS carries a greater risk of progression to invasive disease and is associated more frequently with invasive lobular carcinoma compared with ALH. Mastracci *et al. *demonstrated that somatic alterations in *CDH1 *are a hallmark of LCIS but not ALH [39]. This disparity suggests that mutations that inactivate *CDH1 *can distinguish LNs that are able to progress to invasive disease, explaining our morphological data [39].

## Conclusions

Our findings confirm a frequent association between lobular neoplasia and CCL without atypia, thereby differing from other studies in which the majority of CCL is classified as CCL with atypia or FEA. We also noted a higher frequency of invasive carcinoma, more commonly ILC, that was associated with more developed forms of LN (LCIS).

## List of abbreviations used

ALH: Atypical lobular hyperplasia; BPL: Breast Pathology Laboratory; CCC: Cell change without atypia; CCH: Columnar cell hyperplasia; CCL: Columnar cell lesions; *CDH1: *Gene that encodes E-cadherin; DCIS: Ductal carcinoma *in situ*; DIALH: Ductal involvement by cells of atypical lobular hyperplasia; FEA: Flat epithelial atypia; IDC: Invasive ductal carcinoma; IIL: "indeterminate *in situ *lesions"; ILC: Invasive lobular carcinoma; LCIS: Lobular carcinoma *in situ*; LIN: Lobular intraepithelial neoplasia; LN: Lobular neoplasia; TC: Tubular carcinoma; UFMG: Federal University of Minas Gerais; WHO: World Health Organization.

## Competing interests

The authors declare that they have no competing interests.

## Authors' contributions

*DSG *conceived the study, participated in the histological review, and drafted the manuscript. *DB *participated in the study design, performed the statistical analysis, and drafted the manuscript. *SSP *participated in the design of the study. *HG *participated in design and coordination of the study, participated in the histological review, and drafted and reviewed the manuscript. All authors have read and approved the final manuscript.
